# Date Fruits and Their Products for Almond Beverage Fortification: Assessment of Beverages and Resulting Residues

**DOI:** 10.17113/ftb.64.02.26.9031

**Published:** 2026-06-15

**Authors:** Malek Ben Zid, Nesrine Rokbeni, Nouha M’hiri, Moncef Chouaibi, Nahla Zghonda, Nourhene Boudhrioua

**Affiliations:** 1 Laboratory of Pathophysiology, Food, and Biomolecules, LR17ES03, Higher Institute of Biotechnology of Sidi Thabet, University of Manouba, Ariana 2020, Tunisia; 2 Laboratory of Innovation and Valorization for a Sustainable Food Industry, Higher School of Food Industries of Tunis, Tunisia; 3 Boudjebel VACPA, Km 2 Route de Menzel Bouzelfa, C42, Béni Khalled 8021, Nabeul, Tunisia

**Keywords:** beverages, residues, almonds, date fruits, phenolics, rheological behaviour, sensory properties

## Abstract

**Research background:**

The shift towards plant-based beverages, particularly almond drinks, has increased the need to develop innovative products using fruits and herbal extracts to improve their nutritional and sensory properties. In this study, date palm fruits, Deglet Nour variety (tamr stage) and Besser Helou variety (khalal stage), and two Deglet Nour-derived products (date powder and syrup) were evaluated as fortifying ingredients in almond drinks. The antioxidant and nutritional properties of the recovered solid residues were also evaluated.

**Experimental approach:**

The beverage-making process involved grinding blanched almonds into flour (*w*=12 %), mixing with date products (*w*=20 %), water extraction, filtration, and pasteurisation. Proximate composition, physicochemical properties, phenolic profiles (liquid chromatography-high resolution electrospray ionization mass spectrometry), and antioxidant activity of the raw ingredients, beverages, and recovered residues were analysed using 2,2-diphenyl-1-picrylhydrazyl (DPPH) and Fe(III) reducing antioxidant power (FRAP) assays. In addition, physical stability, rheological behaviour, and sensory attributes of the beverages were evaluated using descriptive analysis and acceptability tests.

**Results and conclusions:**

The addition of date-based ingredients to almonds significantly reduced the extraction yields of beverages, with the reduction being least significant in syrup, moderate in Deglet Nour and Besser Helou varieties, and most significant in date powder. Combining almonds with any form of dates significantly increased carbohydrate and ash mass fraction, energy value and antioxidant activity of beverages, as confirmed by both FRAP and DPPH assays. Phenolics previously present in almonds and dates were found to multiply, while new ones appeared in both drinks and residues, such as caffeic acid. A distinctive rheological profile was observed in beverages fortified with Deglet Nour, date syrup and date powder, characterised by shear thickening at low shear rates and a transition to shear thinning at higher shear rates. Sensory evaluations of enriched drinks revealed that dates provided new colour shades, increased sweetness and mouthfeel viscosity, and improved almond taste perception. Date powder-based drinks received the highest overall acceptability rating, while syrup-based drinks received the lowest. Combining date ingredients with almonds reduced the fat and protein mass fraction of residues while increasing carbohydrate mass fraction. Quinic acid and quercetin were the prevalent phenolic acids and flavonoids in almond residue, while caffeic acid and luteolin were the primary compounds in date-based residues.

**Novelty and scientific contribution:**

This study presents the first comprehensive evaluation of the nutritional, rheological, antioxidant and sensory properties of almond beverages fortified with date-based ingredients. The nutritional and antioxidant properties of the raw ingredients and the produced residues were also assessed. The results suggest that additional phenolics may be generated in fortified almond drinks during processing and show that the resulting date residue is as relevant as potent dietary supplements.

## INTRODUCTION

Research and innovation in food science and technology have witnessed a major shift towards plant-based diets, which are increasingly recommended not only for their health benefits but also for their sustainability and minimal environmental impact (*1*). This change in dietary patterns has led to rapid growth in the global market of plant-based foods, with alternative dairy beverages accounting for the largest share. Within this category, almond milk dominates and is expected to continue growing in the coming years due to increasing consumer preference for gluten-free products (*2*).

To keep pace with this expected growth, recent studies have focused on developing new enriched beverages by combining almond milk with other natural functional ingredients. Some of these studies have examined the effect of almond milk on the bioaccessibility of bioactive compounds derived from added ingredients such as curcumin (*3*), cranberrybush juice (*4*) and rosehip infusion (*5*). Other research has considered the effect of these ingredients on the nutritional and/or functional properties of the final products. For example, a multivitamin supplement for the elderly has been developed by enriching almond milk with a mixture of tempeh milk and vegetable extracts (moringa leaf, beetroot and broccoli). This beverage has been found to provide 100 and 95.25 % of the Recommended Dietary Allowances for vitamin E (0.65 mg/100 mL) and B9 (381.60 mg/100 mL), respectively (*6*). Additionally, fortifying almond milk with one of the following mixtures: carrot juice, honey and stevia; carrot juice, quinoa seed powder, honey and stevia; or banana juice, oat powder, honey and stevia appears to have beneficial effects in preventing metabolic syndrome and the associated hepatic and vascular complications in rats fed a high-fat, high-fructose diet (*7*). Overall, there is strong evidence that adding functional ingredients to almonds improves the nutritional and antioxidant properties of beverages. However, the effects of such ingredients on the sensory profile of the final products, as well as the properties of residues generated after beverage production, have not been addressed.

This study will therefore focus on these aspects by evaluating date palm (*Phoenix dactylifera*) fruits as new ingredients for the production of innovative almond-based drinks. These fruits have been selected for their nutritional value and versatility. Indeed, dates, often called nature’s candies, are composed of digestible sugars, mainly glucose, fructose and sucrose, providing quick and high energy (*8*). They are a rich source of dietary fibre (cellulose, hemicelluloses, pectin, hydrocolloids and lignin) and essential minerals (potassium, calcium, magnesium, phosphorus, sodium, iron, copper, fluorine, sulfur, boron, selenium and zinc) (*9*). They also contain a wide range of bioactive compounds, including carotenoids, phytosterols and phytoestrogens, flavonoids and phenolic acids (*10*).

These compounds, together with dietary fibre, have been associated with a wide range of health-related properties such as antioxidant, prebiotic, antimicrobial, antimutagenic, anti-inflammatory, antitoxic, antihyperlipidaemic, anticancer and gastro-, hepato- and nephron-protective activities (*9*). The chemical composition and sensory properties of dates are influenced, among other factors, by variety, ripening stage and processing method. Depending on the variety, dates can be consumed at three different stages of maturation: khalal or besser (50 % moisture, crisp texture, hard surface, bright or red colour), rutab (30–35 % moisture, partially brown colour) and tamr (10–30 % moisture, soft to hard texture, amber to dark brown colour) (*11*, *12*). Only varieties with low tannin content are suitable for consumption at the besser stage (*13*). In this context, two varieties of Tunisian dates, Deglet Nour and Besser Helou, commonly consumed at two different stages of maturity, tamr and besser respectively, as well as two processed forms, powder and syrup, are evaluated in this work. The selection of these varieties is justified by the fact that Deglet Nour is the main exported variety, known for its long-term preservation at cold temperatures and year-round availability (*14*). Besser Helou, a traditional variety known for its sweetness and astringency, has yet to be valued as a processed fruit. Limited information is available on its nutritional and antioxidant properties at the besser (khalal) stage.

The main aims of this work are: (*i*) to use date palm fruits (Deglet Nour and Besser Helou) and their products (date powder and syrup) to develop innovative fortified almond-based beverages, (*ii*) to investigate the nutritional, physicochemical and physical stability, rheological profiles, and functional and sensory attributes of the developed beverages, and (*iii*) to assess the main nutritional and antioxidant properties of the obtained solid residues for further applications as dietary supplements.

## MATERIALS AND METHODS

### Production of almond beverages and residues

Sweet almonds (*Prunus amygdalus* var. *dulcis*) and Besser Helou dates at the khalal stage were purchased from a local market in Tunis, Tunisia. Fresh Deglet Nour dates at the tamr maturity stage, as well as their processed forms, powder and syrup, were supplied by a date export company, Boudjebel S.A. VACPA (Nabeul, Tunisia).

Almonds were blanched in water at 100 °C for 2 min, peeled, gently wiped with absorbent paper and dried at room temperature for 30 min. For a 1 kg batch, 120 g of almonds (*w*=12 %) were ground into a fine powder using a Thermomix**®** TM6 (Vorwerk, Wuppertal, Germany) at 2000 rpm for 40 s. Subsequently, 100 mL of water were added, and the mixture was blended at 5800 rpm for 1 min. Date ingredients (200 g, *w*=20 %) were then incorporated along with an additional 100 mL of water, and the mixture was blended at 5800 rpm for 1 min. Water was added to reach the total batch mass, and the mixture was filtered through a 200 µm fine mesh filter bag. The filtrate (beverage) was pasteurised at 95 °C for 10 min, cooled to 5 °C, and stored under refrigeration until analysis. Residues remaining in the filter were collected immediately for moisture content and water activity determination, dried at 60 °C in an UN55 oven (Memmert, Büchenbach, Germany) until constant mass, and stored in airtight glass jars at 5 °C until further analyses.

### Yield extraction and distribution of nutrients in beverages and residues

The extraction yield (%) is expressed as the ratio of the beverage mass (kg) to the mass (kg) of the ingredients (almonds, dates and water) (*15*), according to the following equation:


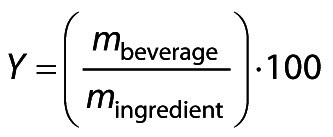
 /1/

#### Proximate composition and energy value determination

The moisture content was determined by drying samples in an oven at 105 °C to a constant mass (*16*). Ash content was assessed by incineration in a muffle furnace (Nabertherm GmbH, Lilienthal, Germany) at 550 °C for 8 h. Total protein content was determined using the Kjeldahl method with a Kjeldahl distillation unit (UDK 129; VELP Scientifica Srl, Usmate, Italy) as described in ISO 665:2020 (*17*) for beverages, and the Kjeldahl method described in JORF (*18*) for raw materials and residues. Fat content was quantified using the Soxhlet extraction method with hexane as the extraction solvent for 6 h in a Soxhlet apparatus (Eurothermal, Venice, Italy). Total carbohydrate content was calculated by subtracting the sum of the mass fractions (in %) of moisture, ash, total fat and total protein from the total sample mass. The energy value was calculated according to the equation below, and expressed in kJ/100 g of sample:


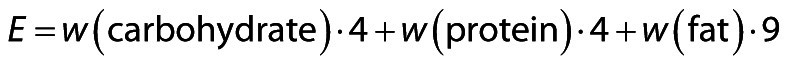
 /2/

### Physicochemical properties

Total soluble solids (TSS) of the samples were determined by the refractometric method according to ISO 2173:2003 (*19*) using a digital refractometer (PAL-3 ATAGO, Japan). The pH was measured according to ISO 1842:1991 (*20*) using a digital pH-meter (PAL-pH; ATAGO, Tokyo, Japan). Titratable acidity was assessed according to ISO 750:1998 (*21*) and expressed in grams of malic acid per 100 g of product. Water activity was determined in triplicate at 20 °C, using a water activity meter (LabMaster-aw neo; Novasina AG, Lachen, Switzerland). The colour attributes of the samples were evaluated by measuring the colour coordinates L*, a* and b* with a colorimeter (PCE-TCR200; PCE Instruments, Tobarra, Spain). Whiteness (WI), yellowness (YI) and browning (BI) indices were used to assess the characteristic colours of almonds (white), Besser Helou (yellow) and Deglet Nour dates, as well as their processed forms (yellow-brown). They were calculated using the following equations (*22*, *23*):


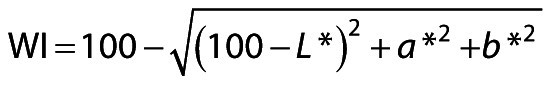
 /3/


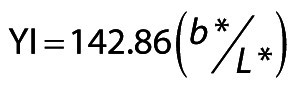
 /4/


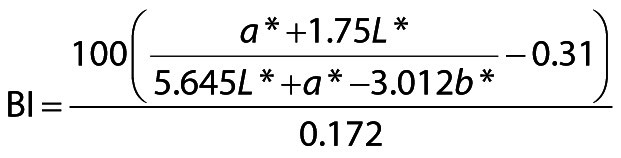
 /5/

The density of beverages was determined using the method reported by Siddeeg *et al.* (*24*) with modifications. A 50-mL flask was washed, dried, cooled in a desiccator and then weighed (*m*_1_). The flask was then filled with the sample beverage and the mass was recorded (*m*_2_). Density was calculated as the ratio of the beverage mass (*m*_2_-*m*_1_) to the known volume (*V*), and expressed in kg/m^3^.

### Antioxidant properties

#### Phenolic extraction and liquid chromatography-high resolution electrospray ionization mass spectrometry analysis

Phenolic compounds were extracted from almonds, dates, beverages and by-products using methanol (minimum 99.8 %, HPLC grade) at a solvent-to-dry material ratio of 1:10 (*m*/*m*) in an ultrasonic cleaning bath (Emmi-60 HC; EMAG AG, Mörfelden-Walldorf, Germany) at 30 °C for 40 min. The sonicated solutions were centrifuged for 5 min at 2711×*g* and 4 °C in a centrifuge (Mikro 220 R; Hettich, Tuttlingen, Germany), then filtered through 0.45-μm Acrodisc® syringe filters (Cytiva, Marlborough, MA, USA) and stored at -20 °C. Samples were analysed using a Shimadzu LCMS-2020 quadrupole mass spectrometer (Kyoto, Japan) equipped with an electrospray ionisation source (ESI) and set to negative ionisation mode. The spectrometer was connected to an ultra-fast liquid chromatography system, which included an LC-20AD XR binary pump system, a SIL-20AC XR autosampler, a CTO-20AC column oven, and a DGU-20A 3R degasser. An Inertsustain C18 column (GL Sciences, Tokyo, Japan) (150 mm×3 mm, 3 μm) was used for analysis. The mobile phase consisted of 0.02 % acetic acid in aqueous solution of acetonitrile, with a 10-minute acquisition time. The mobile phase flow rate was 0.4 mL/min, the column temperature was maintained at 40 °C, and the injection volume was 5 µL. Spectra were collected in selected ion monitoring (SIM) mode and analysed with Shimadzu LabSolutions LC-MS. Phenolic substances were identified by comparing retention times and mass spectra to chemical standards of >98 % purity from Sigma-Aldrich, Merck (St Louis, MO, USA). The results were expressed as mg of phenolic compound per 100 g of sample (*25*, *26*).

### Antioxidant activity assays (DPPH and FRAP)

The antioxidant potential of the extracts was evaluated using two spectrophotometric methods: the 2,2-diphenyl-1-picrylhydrazyl (DPPH) radical scavenging assay and the Fe(III) reducing antioxidant power (FRAP) assay. Absorbance was measured with a UV-Vis spectrophotometer (PEAK Instruments, Houston, TX, USA).

DPPH radical scavenging activity was determined according to the method described by Ben Abdallah *et al.* (*27*). Briefly, 400 µL of the sample were mixed with 2.4 mL of methanolic DPPH solution (0.02 mg/mL). The mixture was vortexed and kept in the dark at room temperature for 20 min, after which absorbance was measured at 517 nm.

The FRAP assay was conducted following the method of Benzie and Strain (*28*) with slight modifications. The FRAP reagent was freshly prepared by mixing 0.2 M acetate buffer (pH=3.6), 10 mM 2,4,6-tripyridyl-*s*-triazine (TPTZ) solution in 40 mM HCl, and 20 mM FeCl_3_·6H_2_O in a volume ratio of 10:1:1. Then, 100 µL of the sample were added to 2 mL of FRAP reagent and incubated at 37 °C for 30 min in the dark. The increase in absorbance was measured at 593 nm.

Results for both assays were expressed as mg Trolox equivalents (TE) per 100 g of sample.

### Rheological characterisation

The rheological properties of the beverages were measured using a rotary viscometer (Rheometric RM180; Rheomat, Caluire, France) equipped with a coaxial cylinder geometry. Steady shear tests were carried out over a shear rate range of 10–500 s^−1^ at a controlled temperature of 25 °C. Apparent viscosity (*η*_app_) was recorded as a function of shear rate (𝛾˙), and the flow behaviour of each sample was modelled using the Ostwald–de Waele (power-law) equation (*29*):


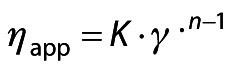
 /6/

where *η*_app_ is the apparent viscosity (Pa·s), 𝛾˙ is the shear rate (s^−1^), *K* is the consistency index (Pa·s^N^), and *n* is the flow behaviour index (dimensionless). The goodness of fit of the model was evaluated by regression analysis, and the coefficient of determination (R^2^) was used to assess model adequacy.

### Physical stability

Physical stability of beverages was assessed by determining the sedimentation index under centrifugation, as described by Su *et al.* (*30*) with minor modifications, and by monitoring phase separation at 4 °C. The sedimentation index was determined by centrifuging 30 g of beverage in 50-mL centrifuge tubes at 4427×*g* for 10 min using a FRONTIER™ FC5706 centrifuge (OHAUS®, Nänikon, Switzerland). The supernatant was carefully discarded, and the mass of the sediment was recorded. The sedimentation index was calculated as the ratio of the sediment mass (g) to the initial beverage mass (g), as shown in the following equation:


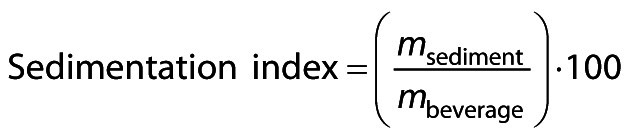
 /7/

Stability at rest was also evaluated at 4 °C by transferring samples into 15-mL sealed centrifuge tubes, leaving them undisturbed, and measuring the heights (in mm) of the different phases (sediment, intermediate liquid, and top supernatant) after 0, 24, 96 and 240 h. The separation kinetics was expressed using a phase ratio calculated as a function of time, as shown in the following equation:


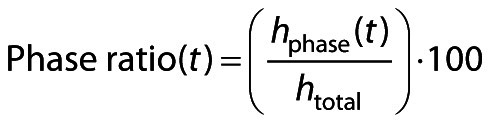
 /8/

where *h*_phase_(*t*) is the height (in mm) of the considered phase at time *t*, and *h*_total_ is the total height of the sample (mm).

### Sensory evaluation

The sensory profile of the beverages was evaluated by internally recruited panellists (7 women and 3 men aged between 26 and 45) from the Higher Institute of Biotechnology of Sidi Thabet, Sidi Thabet, Tunisia, who were trained in accordance with ISO 8586:2023 (*31*) to ensure consistency and reliability in descriptive sensory analysis. Training focused on attribute definition, consensus building, and repeatability under the supervision of the panel leader. Nine descriptors were selected for the assessment of appearance (brown colour and visual viscosity), odour (almond odour, date odour), flavour (almond flavour, date flavour), taste (sweetness and acidity), and mouthfeel (viscosity mouthfeel), using a 7-point intensity scale (1=not perceived, 7=extremely strong intensity). Beverages were evaluated after one day of processing and cold storage at 5 °C. Participants received all samples at once after homogenisation in 60-mL clear plastic drinking cups with lids, coded with 3-digit numbers in a random order at 20 °C.

The hedonic test was performed by 30 untrained panellists (21 women and 9 men aged between 22 and 53). They were asked to score their liking of colour, odour, viscosity, taste and overall attributes (*7*) using a 7-point hedonic scale from “dislike extremely” to “like extremely”. Panellists received mineral water and soft unsalted bread for palate cleansing between tastings in all sensory sessions.

### Statistical analysis

Analysis of variance (ANOVA) and Tukey’s *post-hoc* test were used to compare the different attributes of raw materials, beverages, and residues. Significance was set at p<5 %. Principal component analysis (PCA) and Pearson correlation test were applied to interpret sensory data and correlate them with nutritional, antioxidant and physicochemical properties. All statistical tests were performed using XLSTAT software v. 2019 (*32*).

## RESULTS AND DISCUSSION

### Physicochemical and functional profile of almonds and date products

#### Compositional and physicochemical attributes of almonds and date products

The proximate composition of the different ingredients (Deglet Nour, Besser Helou, date powder and syrup, blanched almonds), given in Table 1 on a wet mass basis, showed that blanched almonds contained (6.6±0.2) % moisture, (47.08±0.01) % fat, (26.66±0.05) % protein, (16.5±0.2) % carbohydrates and (3.18±0.01) % ash. They provided 1.5–4-fold higher caloric values than date-based ingredients, which were characterised by high carbohydrate mass fraction (37.8–88.78 %) and low mass fractions of fat (0.02–0.28 %), protein (1.58–3.24 %), and ash (1.02–2.41 %). These ingredients differed from each other according to date variety, ripening stage and processing method. On average, moisture made up (59.4±0.3) % of Besser Helou mass, followed by carbohydrates ((37.8±0.2) %), protein ((1.58±0.03) %), ash ((1.02±0.01) %) and fat ((0.28±0.01) %). By contrast, three-quarters of Deglet Nour date total mass was carbohydrates ((75.11±0.08) %), with moisture mass fraction accounting for only one-fifth ((21.0±0.1) %). They contained less fat ((0.07±0.01) %) and more protein ((2.16±0.05) %) and ash ((1.68±0.02) %) than Besser Helou. These findings are consistent with the established fact that moisture content decreases from 50–60 % to about 10–25 % as sugars accumulate during the ripening of date fruits from the khalal to tamr stage. These changes in moisture and carbohydrate content prevent the fruits from fermenting, thereby enabling long-term storage (*33*). The powder contained less moisture ((5.51±0.08) %), and consequently more protein ((3.24±0.02) %), ash ((2.41±0.01) %), carbohydrates ((88.78±0.09) %) and energy ((1543±1) kJ/100 g) than fresh dates. It was also richer in nutrients than the syrup, from which some insoluble compounds were partially eliminated by filtration during the manufacturing process. Almonds had the highest pH (5.82±0.02) and the lowest titratable acidity (TA; (0.34±0.02) %) and total soluble solids (TSS; (26.00±0.35) g/100 g), compared with date-based ingredients, which showed a more acidic pH range (4.20±0.01–5.07±0.02) and higher TA ((0.36±0.02)–(0.71±0.03) %) and TSS values ((37.0±0.4)–(90.0±0.6) g/100 g). These results are due to the fact that date fruits contain much more organic acids and soluble sugars than almonds. Almonds contained small mass fractions of soluble sugars, including 3.95 % sucrose, 0.17 % glucose, 0.11 % fructose, with negligible amounts of other monosaccharides (<0.1 %) and sugar alcohols (*34*).

**Table 1:** Nutritional and physicochemical properties of raw ingredients, beverages, and their corresponding residues

Ingredient	Blanched almonds	Besser Helou	Deglet Nour	Syrup	Powder
*w*(moisture)/%	(6.6±0.2)^b^	(59.4±0.3)^e^	(21.0±0.1)^c^	(29.58±0.04)^d^	(5.51±0.08)^a^
*w*(ash)/%	(3.18±0.01)^e^	(1.02±0.01)^a^	(1.68±0.02)^b^	(1.750±0.001)^c^	(2.41±0.01)^d^
*w*(fat)/%	(47.08±0.01)^d^	(0.28±0.01)^c^	(0.07±0.01)^b^	(0.02±0.01)^c^	(0.07±0.01)^b^
*w*(protein)/%	(26.66±0.05)^d^	(1.58±0.03)^a^	(2.16±0.05)^b^	(1.61±0.01)^a^	(3.24±0.02)^c^
*w*(carbohydrate)/%	(16.5±0.2)^a^	(37.8±0.2)^b^	(75.11±0.08)^d^	(67.04±0.04)^c^	(88.78±0.09)^e^
*E*/(kJ/100 g)	(2494±4)^e^	(669±4)^a^	(1296±2)^c^	(1149.6±0.6)^b^	(1543±1)^d^
*w*(TSS)/(g/100 g)	(26.0±0.4)^a^	(37.0±0.4)^b^	(74.400±0.001)^c^	(73.8±1.0)^c^	(90.0±0.6)^d^
pH	(5.82±0.02)^e^	(4.86±0.02)^c^	(5.07±0.02)^d^	(4.20±0.01)^a^	(4.67±0.01)^b^
TA/%	(0.34±0.02)^a^	(0.36±0.02)^a^	(0.71±0.03)^c^	(0.60±0.03)^b^	(0.57±0.02)^b^
*a*_w_	(0.742±0.002)^c^	(0.9336±0.0005)^e^	(0.6521±0.0003)^b^	(0.7322±0.0005)^d^	(0.282±0.002)^a^
Beverage	A	ABH	ADN	AS	AP
*Y*_extraction_/%	(89.7±1.0)^e^	(79.7±1.4)^bc^	(80.8±0.4)^c^	(88.4±1.8)^d^	(76.0±2.0)^a^
*w*(moisture)/%	(92.86±0.04)^d^	(87.40±0.02)^c^	(81.31±0.03)^b^	(80.6±1.0)^b^	(79.02±0.06)^a^
*w*(ash)/%	(0.22±0.02)^a^	(0.30±0.01)^b^	(0.42±0.01)^c^	(0.57±0.04)^d^	(0.58±0.01)^d^
*w*(fat)/%	(3.98±0.08)^e^	(3.80±0.01)^d^	(3.54±0.04)^c^	(2.68±0.04)^a^	(2.98±0.03)^b^
*w*(protein)/%	(2.17±0.03)^b^	(2.07±0.03)^a^	(2.38±0.02)^d^	(2.17±0.03)^b^	(2.31±0.01)^c^
*w*(carbohydrate)/%	(0.76±0.03)^a^	(6.43±0.04)^b^	(12.35±0.06)^c^	(14.4±0.4)^d^	(15.11±0.09)^d^
*E*/(kJ/100 g)	(199.1±1.8)^a^	(285.2±0.4)^b^	(379.9±1.0)^c^	(377.5±8.2)^c^	(403.8±0.7)^d^
*w*(TSS)/(g/100 g)	(4.6±0.2)^a^	(8.9±0.4)^b^	(15.8±0.1)^c^	(16.8±1.4)^c^	(18.5±0.2)^d^
pH	(6.3±0.2)^d^	(6.0±0.1)^c^	(5.500±0.001)^b^	(5.07±0.06)^a^	(5.3±0.1)^ab^
TA/%	(0.42±0.07)^a^	(2.32±0.07)^c^	(1.49±0.07)^b^	(2.95±0.07)^d^	(2.88±0.08)^d^
*a*_w_	(0.966±0.004)^ab^	(0.9701±0.0006)^b^	(0.959±0.001)^a^	(0.962±0.001)^ab^	(0.965±0.005)^ab^
*ρ*/(g/L)	(978.1±0.9)^a^	(1009.6±0.4)^b^	(1034.3±0.6)^d^	(1029.94±0.05)^c^	(1054.8±0.8)^e^
Residue	A	ABH	ADN	AS	AP
*w*(moisture before drying)/%	(58.60±0.06)^b^	(71.9±0.2)^e^	(66.6±0.2)^d^	(56.4±0.2)^a^	(63.1±0.1)^c^
*a*_w_ before drying	(0.97±0.01)^a^	(0.96±0.01)^a^	(0.9701±0.0007)^a^	(0.9645±0.0009)^a^	(0.95±0.01)^a^
*w*(moisture after drying/%	(5.76±0.04)^a^	(9.09±0.08)^b^	(9.83±0.03)^d^	(9.61±0.01)^c^	(9.81±0.01)^d^
*w*(ash)/%	(2.74±0.03)^b^	(2.66±0.01)^b^	(2.3±0.1)^ab^	(2.54±0.06)^b^	(1.8±0.4)^a^
*w*(fat)/%	(58.39±0.01)^e^	(30.13±0.01)^c^	(24.86±0.01)^b^	(41.93±0.01)^d^	(25.21±0.01)^a^
*w*(protein)/%	(22.47±0.03)^e^	(8.45±0.02)^a^	(8.74±0.06)^b^	(14.40±0.08)^d^	(10.08±0.05)^c^
*w*(carbohydrate)/%	(10.65±0.04)^a^	(49.67±0.09)^c^	(54.3±0.1)^d^	(36.52±0.07)^b^	(53.0±0.4)^d^
*E*/(kJ/100 g)	(2753±1)^e^	(2107±1)^c^	(1991±2)^a^	(2347±1)^d^	(2006±7)^b^
*w*(TSS)/(g/100 g)	(2.4±1.6)^a^	(10.0±0.4)^c^	(7.2±0.6)^b^	(9.8±0.4)^c^	(13.2±0.6)^d^
pH	(5.77±0.20)^b^	(5.1±0.3)^a^	(5.3±0.1)^ab^	(5.4±0.3)^ab^	(4.9±0.3)^a^
TA/%	(0.27±0.02)^ab^	(0.28±0.02)^a^	(0.24±0.02)^a^	(0.23±0.03)^a^	(0.36±0.02)^b^
*a*_w_	(0.5988±0.0004)^a^	(0.5990±0.0001)^a^	(0.6000±0.0001)^a^	(0.5996±0.0001)^a^	(0.6001±0.0002)^a^

Data are expressed on a fresh mass basis as mean value±standard deviation, *N*=3. Mean values followed by different letters in superscript within columns indicate significant differences at p≤0.05 according to Tukey’s test. TSS=total soluble solutes, TA=titratable acidity, *a*_w_=water activity, A=blanched almonds, BH=Besser Helou, DN=Deglet Nour, S=date syrup, P=date powder

### Functional attributes of almonds and date products

The identified phenolic compounds in the methanolic extracts of the different ingredients (Deglet Nour, Besser Helou, date powder and syrup, blanched almonds) are listed in Table 2. The extract of blanched almonds showed the presence of four cinnamic acid derivatives (quinic acid, 1,3-di-O-caffeoylquinic acid, chlorogenic acid and rosmarinic acid) and five flavonoids: one flavonol (quercetin), three flavones (apigenin-7-O-glucoside, luteolin and cirsiliol) and one flavanone (naringenin). Quinic acid and apigenin-7-O-glucoside were the major compounds among the phenolic acids and flavonoids. With the exception of chlorogenic acid, luteolin and naringenin, all other phenolics were newly identified in almond kernels. Chlorogenic acid and luteolin have been detected in whole kernels of 20 Serbian varieties (*35*), with mass fractions ranging from 0.098 to 2.103 mg/100 g and from 0.034 to 0.624 mg/100 g respectively, which agrees with our findings of (1.526±0.005) mg/100 g of chlorogenic acid and (0.498±0.001) mg/100 g of luteolin. Naringenin has been found in eight varieties of blanched Californian almonds (*36*) and in the Serbian whole almonds mentioned earlier, with mass fractions ranging from 0.009 to 1.574 mg/100 g (*35*), consistent with our finding of (0.958±0.001) mg/100 g.

**Table 2:** Identified phenolic compounds and antioxidant properties of raw ingredients, beverages, and their corresponding residues

Ingredient	Blanched almonds	Besser Helou	Deglet Nour	Syrup	Powder
	*w*(phenolic acid)/(mg/100 g)				
Quinic acid	40.45±0.05	ND	ND	ND	ND
Chlorogenic acid	(1.526±0.005)^a^	(1.596±0.002)^b^	(5.002±0.002)^c^	(8.981±0.001)^e^	(5.926±0.006)^d^
1,3-di-O-Caffeoylquinic acid	(19.857±0.003)^b^	(13.620±0.001)^a^	(44.850±0.002)^c^	(51.582±0.001)^e^	(47.923±0.002)^d^
*trans-*Ferulic acid	ND	ND	ND	ND	1.592±0.002
o-Coumaric acid	ND	(1.701±0.001)^c^	(1.560±0.002)^b^	ND	ND
Rosmarinic acid	(0.701±0.001)^a^	(7.320±0.001)^b^	(3.939±0.001)^c^	(10.594±0.002)^d^	(17.897±0.007)^e^
Salvianolic acid	ND	(0.663±0.001)^a^	(1.341±0.002)^c^	ND	(1.257±0.002)^b^
Total phenolic acids	(62.53±0.05)^c^	(24.874±0.001)^a^	(61.692±0.003)^b^	(71.157±0.002)^d^	(74.595±0.008)^e^
	*w*(flavonoid)/(mg/100 g)				
Naringin	ND	1.777±0.001	ND	ND	ND
Apigenin-7-O-glucoside	(2.676±0.001)^a^	(3.472±0.002)^b^	(7.435±0.001)^d^	(11.330±0.001)^e^	(5.631±0.001)^c^
Quercetin	(0.23±0.03)^a^	(0.363±0.001)^b^	(0.356±0.001)^b^	(0.368±0.002)^b^	(0.576±0.001)^c^
Naringenin	(0.958±0.001)^b^	ND	ND	ND	ND
Luteolin	(0.498±0.001)^b^	(13.972±0.001)^c^	(5.786±0.002)^b^	ND	(3.656±0.003)^a^
Cirsiliol	(0.157±0.002)^a^	(0.497±0.001)^b^	(4.176±0.002)^d^	(3.368±0.002)^c^	(7.871±0.002)^e^
Acacetin	ND	ND	0.192±0.002	ND	ND
Total flavonoids	(4.52±0.03)^a^	(19.480±0.002)^e^	(17.945±0.004)^d^	(15.068±0.003)^b^	(17.733±0.002)^c^
Total identified phenolics/(mg/100 g)	(67.06±0.06)^b^	(44.354±0.002)^a^	(79.637±0.007)^c^	(86.225±0.002)^d^	(92.33±0.01)^e^
DPPH as TE/(mg/100 g)	(23.8±1.3)^a^	(23.47±0.08)^a^	(45.27±0.09)^b^	(49.4±0.1)^c^	(53.5 ±0.1)^d^
FRAP as TE/(mg/100 g)	(33.6±1.7)^a^	(65.3±1.5)^c^	(52.0±2.9)^b^	(39.7±4.3)^a^	(171.0±0.4)^d^
Beverage	A	ABH	ADN	AS	AP
	*w*(phenolic acid)/(mg/100 g)				
Quinic acid	(149.96±0.06)^e^	(143.02±0.02)^d^	(46.61±0.01)^b^	(59.687±0.006)^c^	(35.508±0.007)^a^
Chlorogenic acid	(2.659±0.008)^c^	(2.559±0.001)^b^	(3.079±0.001)^d^	(7.107±0.001)^e^	(0.184±0.006)^a^
Caffeic acid	ND	(80.33±0.02)^b^	(171.42±0.02)^c^	(69.310±0.001)^a^	ND
1,3-di-O-caffeoylquinic acid	(9.01±0.01)^a^	(9.864±0.003)^b^	(62.537±0.001)^e^	(43.927±0.001)^d^	(35.943±0.002)^c^
*o*-Coumaric acid	ND	(0.222±0.001)^a^	(1.967±0.001)^c^	(1.079±0.001)^b^	ND
Rosmarinic acid	(1.126±0.001)^a^	(25.244±0.001)^e^	(18.317±0.001)^c^	(18.679±0.001)^d^	(7.628±0.001)^b^
Salvianolic acid	(4.065±0.001)^b^	(1.106±0.001)^a^	ND	ND	ND
Total phenolic acids	(166.82±0.06)^b^	(262.34±0.04)^d^	(303.93±0.03)^e^	(199.78±0.01)^c^	(74.26±0.01)^a^
	*w*(flavonoid)/(mg/100 g)				
Rutin	ND	ND	ND	0.136±0.001	ND
Hyperoside	(0.435±0.001)^a^	(1.145±0.001)^c^	(1.012±0.001)^b^	ND	ND
Naringin	ND	(2.107±0.001)^b^	(1.382±0.001)^a^	ND	ND
Quercitrin	ND	ND	(0.982±0.001)^a^	(2.084±0.001)^b^	ND
Apeginin-7-O-glucoside	(0.683±0.001)^b^	(0.345±0.002)^a^	(5.762±0.001)^d^	(7.942±0.001)^e^	(4.579±0.001)^c^
Quercetin	(0.268±0.001)^a^	(0.281±0.001)^b^	(0.487±0.001)^d^	(0.335±0.001)^c^	(0.612±0.001)^e^
Naringenin	ND	0.382±0.001	ND	ND	ND
Luteolin	ND	(2.462±0.001)^a^	(4.468±0.001)^c^	ND	(4.200±0.002)^b^
Cirsiliol	ND	(0.216±0.001)^a^	(1.277±0.002)^d^	(0.745±0.002)^b^	(1.006±0.002)^c^
Total flavonoids	(1.386±0.001)^a^	(6.938±0.004)^b^	(15.370±0.001)^e^	(11.242±0.001)^d^	(10.397±0.002)^c^
Total identified phenolics	(168.21±0.06)^b^	(269.28±0.04)^d^	(319.30±0.03)^e^	(211.02±0.01)^c^	(84.660±0.009)^a^
DPPH as TE/(mg/100 g)	(11.6±2.0)^a^	(26.8±2.0)^b^	(31.2±0.4)^b^	(48.5±2.1)^c^	(47.5±2.5)^c^
FRAP as TE/(mg/100 g)	(1.5±0.2)^a^	(11.4±1.7)^b^	(12.9±0.7)^bc^	(15.2± 0.2)^c^	(39.56±0.06)^d^
Residue	A	ABH	ADN	AS	AP
	*w*(phenolic acid)/(mg/100 g)				
Quinic acid	(201.451±0.002)^e^	(44.115±0.001)^d^	(40.014±0.002)^c^	(19.000±0.001)^a^	(21.078±0.001)^b^
Chlorogenic acid	(12.611±0.001)^e^	(7.679±0.002)^c^	(7.891±0.001)^d^	(6.058±0.001)^a^	(7.273±0.002)^b^
Caffeic acid	(47.841±0.001)^a^	(53.439±0.001)^b^	(57.057±0.002)^d^	(54.138±0.001)^c^	(67.899±0.001)^e^
1,3-di-O-caffeoylquinic acid	(14.833±0.002)^a^	(42.334±0.001)^d^	(30.742±0.001)^b^	(33.034±0.001)^c^	(33.279±0.001)^c^
*trans*-Ferulic acid	(0.343±0.001)^a^	(0.898±0.001)^e^	(0.530±0.001)^d^	(0.392±0.001)^c^	(0.359±0.001)^b^
*o*-Coumaric acid	ND	(4.982±0.001)^d^	(1.834±0.001)^a^	(4.965±0.005)^c^	(4.084±0.001)^b^
Rosmarinic acid	(8.046±0.002)^a^	(21.451±0.001)^b^	(25.756±0.002)^d^	(28.543±0.002)^e^	(22.776±0.002)^c^
Salvianolic acid	(9.540±0.001)^e^	(6.695±0.001)^d^	(4.558±0.001)^c^	(2.946±0.001)^b^	(2.243±0.001)^a^
Total phenolic acids	(294.7±0.6)^e^	(181.593±0.008)^d^	(168.382±0.003)^c^	(149.076±0.006)^a^	(158.992±0.002)^b^
	*w*(flavonoid)/(mg/100 g)				
Hyperoside	(0.510±0.001)^a^	(2.234±0.002)^e^	(1.007±0.001)^b^	(1.819±0.001)^d^	(1.641±0.001)^c^
Naringin	(1.466±0.003)^a^	(4.215±0.002)^b^	ND	ND	ND
Quercitrin	ND	ND	ND	(1.984±0.001)^a^	(2.100±0.001)^b^
Apeginin-7-O-glucoside	(0.967±0.001)^b^	(0.859±0.001)^a^	(0.997±0.001)^c^	(3.273±0.002)^e^	(2.318±0.001)^d^
Quercetin	(1.994±0.001)^b^	(3.599±0.001)^d^	(2.954±0.001)^c^	(1.753±0.002)^a^	(4.666±0.001)^e^
Naringenin	(1.235±0.001)^d^	(1.826±0.001)^e^	(0.982±0.001)^b^	(0.763±0.001)^a^	(1.154±0.001)^c^
Luteolin	ND	(11.417±0.001)^b^	(45.836±0.002)^d^	(5.517±0.001)^a^	(27.719±0.002)^c^
Cirsiliol	(0.642±0.001)^a^	(9.025±0.001)^e^	(8.036±0.002)^c^	(2.893±0.006)^b^	(8.419±0.001)^d^
Total flavonoids	(6.81±0.03)^a^	(33.175±0.003)^c^	(59.810±0.003)^e^	(17.997±0.006)^b^	(48.817±0.002)^d^
Total identified phenolics	(301.5±0.6)^e^	(214.768±0.006)^c^	(228.194±0.003)^d^	(167.074±0.009)^a^	(207.009±0.001)^b^
DPPH as TE/(mg/100 g)	(27.7±3.0)^a^	(46.0±2.5)^b^	(52.9±0.6)^c^	(50.6±0.5)^bc^	(53.3±0.3)^c^
FRAP as TE/(mg/100 g)	(14.0±2.8)^b^	(12.3±0.1)^a^	(36.1±6.0)^c^	(25.4±1.5)^bc^	(52.6±5.0)^d^

Data are expressed on a fresh mass basis as mean value±standard deviation, *N*=3. Mean values followed by different letters in superscript within columns indicate significant differences at p≤0.05 according to Tukey’s test. ND=not detected, A=blanched almonds, BH=Besser Helou, DN=Deglet Nour, S=date syrup, P=date powder

The date-based ingredients shared common phenolic compounds with almonds, except for quinic acid and naringenin, which appeared specific to almonds, and luteolin, which was not detected in syrup. They also contained other compounds, including *trans*-ferulic acid, *o*-coumaric acid, salvianolic acid, naringin and acacetin. *Trans*-ferulic acid was found only in the powder extract, while *o*-coumaric acid was detected in both fresh dates of Deglet Nour and Besser Helou varieties. Salvianolic acid was present in all extracts of date products except syrup. Naringin was found only in Besser Helou, while acacetin was detected only in Deglet Nour. Similar findings have been reported for fresh Deglet Nour, indicating the presence of acacetin, as well as luteolin, quercetin and cirsiliol (*37*). Quantitatively, the common phenolics were higher in date-based products than in almonds, except for 1,3-di-O-caffeoylquinic acid, which was lower in Besser Helou than in almonds. The comparison of individual phenolic compounds in date-based ingredients showed significant differences depending on variety and processing technique. Besser Helou contained the highest mass fractions of *o*-coumaric acid and luteolin, while Deglet Nour showed the highest mass fraction of salvianolic acid. The syrup was characterised by the highest mass fractions of chlorogenic acid, 1,3-di-O-caffeoylquinic acid and apigenin 7-O-glucoside. Rosmarinic acid, quercetin and cirsiliol were the most abundant ingredients in date powder. In all ingredients, the mass fractions of phenolic acids were higher than those of flavonoids. The highest mass fraction of phenolic acids was found in the powder extract (about 74.6 mg/100 g), while the lowest was observed in the Besser Helou extract (24.87 mg/100 g). All date-based products had approx. 3-4 times more flavonoids than blanched almonds. Accordingly, the lowest total flavonoid mass fraction was observed in almonds. Besser Helou showed the highest total flavonoid mass fraction due to its high luteolin content, followed by Deglet Nour, date powder and syrup. However, considering the total phenolic mass fraction, date powder was the richest, followed by syrup, Deglet Nour, almonds and Besser Helou. This may be due to the high dry matter content of processed fruits (powder and syrup) compared to raw ones (Deglet Nour), as well as Deglet Nour at the tamr stage compared to that of Besser Helou at the khalal stage. Date powder exhibited the highest antioxidant activity in both DPPH and FRAP assays, with values significantly higher than those of all other samples, confirming its high amount of redox-active compounds. In contrast, almonds and Besser Helou showed the lowest DPPH radical-scavenging activity, indicating a limited ability to neutralise free radicals through hydrogen or electron donation. The FRAP assay revealed a different pattern: almonds and date syrup showed the lowest reducing power, suggesting weaker Fe^3+^-reducing capacity. Deglet Nour exhibited intermediate antioxidant activities between these extremes, although its relative position varied slightly between assays.

#### Colour characteristics of almonds and date products

**Fig. S1:**
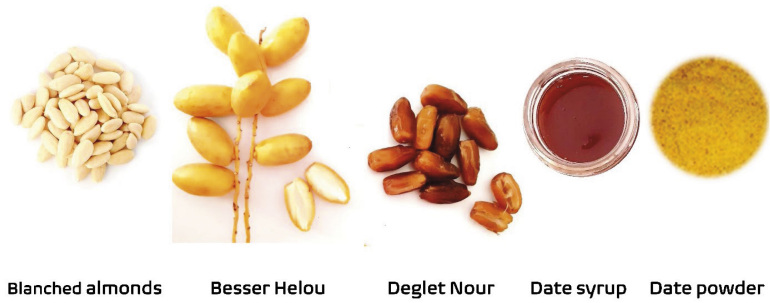
Visual aspect of ingredients

The blanched almonds, off-white in colour (Fig. S1), showed the highest whiteness index (WI) value and the lowest browning index (BI) value (Table 3). The yellow colour of Besser Helou, indicated by the highest yellowness index (YI) value of 60.6±4.3, could be attributed to the predominance of luteolin, while the brown colour of Deglet Nour, indicating full ripening, could be associated with the mass fraction of dactyliferic acid (5-O-caffeoylshikimic acid) (*38*). The deep dark brown colour of syrup, with a high browning index of 96.6±3.5, may be due to the formation of melanoidins through Maillard reaction (*39*). The date powder had a sandy colour, with WI, YI and BI mean values of 50.4, 49.0 and 56.1, respectively.

**Table 3:** Colour properties of raw ingredients, beverages, and their corresponding residues

Ingredient	Blanched almonds	Besser Helou	Deglet Nour	Syrup	Powder
L*	(75.9±0.4)^e^	(62.3±1.9)^d^	(46.07±0.09)^b^	(4.2±0.2)^a^	(55.8±0.3)^c^
a*	(2.6± 0.6)^a^	(4.2±0.1)^a^	(8.1±0.4)^c^	(8.0±0.2)^c^	(11.8±0.4)^d^
b*	(15.0±0.6)^c^	(26.4±1.1)^e^	(12.6± 0.3)^b^	(-0.17±0.04)^a^	(19.2±0.3)^c^
WI	(71.5±0.7)^e^	(53.8±2.2)^d^	(44.0±0.1)^b^	(3.9±0.5)^a^	(50.4±0.2)^c^
YI	(28.2±1.2)^b^	(60.6±4.3)^e^	(39.1±1.1)^c^	(-5.1±1.6)^a^	(49.0±0.7)^d^
BI	(23.8±1.6)^a^	(58.2±5.1)^d^	(43.8±1.1)^b^	(96.6±3.6)^e^	(56.1±0.5)^c^
Beverage	A	ABH	ADN	AS	AP
L*	(96.0±1.8)^d^	(75.4±2.8)^c^	(66.7±1.5)^b^	(65.1±1.2)^b^	(50.5±1.9)^a^
a*	(2.3±0.2)^a^	(4.0±1.0)^ab^	(9.3±2.0)^c^	(6.9±0.5)^bc^	(15.5±0.2)^d^
b*	(8.6±0.6)^a^	(12.4±0.5)^b^	(13.1±1.0)^b^	(20.9±1.2)^d^	(16.2±0.2)^c^
WI	(90.2±0.6)^e^	(72.0±2.5)^d^	(63.0±0.6)^c^	(58.7±0.7)^b^	(47.0±3.0)^a^
YI	(12.7±0.8)^a^	(23.4±1.2)^b^	(28.1±1.6)^c^	(45.9±2.2)^d^	(45.9±1.6)^d^
BI	(10.8±0.5)^a^	(21.6±1.2)^b^	(31.3±3.0)^c^	(45.4±1.7)^d^	(59.7±2.7)^e^
Residue	A	ABH	ADN	AS	AP
L*	(81.5±5.0)^d^	(80.3±1.9)^d^	(70.6±2.3)^c^	(46.1±1.5)^a^	(56.3±1.1)^b^
a*	(1.4±0.6)^a^	(2.6±1.8)^ab^	(2.3±1.5)^ab^	(9.8±3.7)^c^	(7.6±1.7)^bc^
b*	(2.4±0.7)^a^	(4.1±2.0)^a^	(6.0±2.3)^a^	(14.9±0.4)^b^	(18.7±0.8)^b^
WI	(81.2±4.9)^d^	(79.6±1.8)^d^	(69.8±2.4)^c^	(43.1±1.1)^a^	(51.8±1.1)^b^
YI	(4.2±1.2)^a^	(7.2±3.4)^a^	(12.1±4.7)^a^	(46.3±2.3)^b^	(47.5±1.9)^b^
BI	(4.0±0.3)^a^	(7.4±0.8)^a^	(10.9±5.0)^a^	(53.4±3.5)^b^	(49.1±1.8)^b^

Data are expressed as mean value±standard deviation, *N*=3. Mean values followed by different letters in superscript within columns indicate significant differences at p≤0.05 according to Tukey’s test. L*=lightness, a*=redness, b*=yellowness, WI=whiteness index, YI=yellowness index, BI=browning index, A=blanched almonds, BH=Besser Helou, DN=Deglet Nour, S=date syrup, P=date powder

### Physicochemical, functional and sensory attributes of formulated beverages

#### Compositional and physicochemical attributes of beverages

As shown in Table 1, the extraction yields of beverages were considerably reduced when date-based ingredients were added to almonds. However, this reduction was less significant with syrup ((88.9±1.8) %), moderate with Deglet Nour ((80.8±0.4) %) and Besser Helou ((79.7±1.4) %), and more significant with date powder ((76.0±2.0) %). This may be explained by the fact that syrup dissolved more readily than the other ingredients. Fresh dates contain hydrophobic substances, mainly insoluble fibre (lignin) and waxes (*40*, *41*), which cannot be extracted with water, while the dehydrated form of date powder favoured water absorption simultaneously with the dissolution of soluble substances during the process. A mass of 1 g of Deglet Nour powder could hold (2.0±0.2) mL of water (*42*).

Based on the proximate composition of beverages given in Table 1, the almond drink contains 92.86 % moisture, 3.98 % fat, 2.17 % protein, 0.22 % ash and 0.76 % carbohydrates. The energy value was 199.1 kJ/100 g. Adding date products to almonds significantly decreased the moisture and fat contents of the beverages while increasing the carbohydrate and ash contents. Besser Helou, the ingredient with the lowest dry matter, caused the least changes, showing a drop in moisture, fat and protein to about 87.40, 3.80 and 2.07 %, respectively, and an increase in carbohydrates and ash to about 6.43 and 0.30 %, respectively. Due to its highest dry matter mass fraction, date powder caused the greatest drop in moisture content of the beverage, reaching (79.02±0.06) %. Along with syrup, it provided the largest enrichment in ash and carbohydrates, showing increases of 2.5 times for ash (0.30–0.58 %) and about 19–20 times for carbohydrates (6.43–15.11 %). The protein mass fraction increased only with the incorporation of Deglet Nour ((2.38±0.02) %) and powder ((2.31±0.01) %). It decreased when Besser Helou was added ((2.07±0.03) %) and remained unchanged with syrup ((2.17±0.03) %). Similar effects have been reported for palmyrah fruit pulp when mixed with coconut beverage, with a decrease in mass fractions of moisture (from 85.06 to 80.47 %) and fat (from 5.61 to 1.50 %), and an increase in mass fractions of ash (from 0.26 to 3.77 %), protein (from 1.27 to 2.80 %) and carbohydrate (from 22.19 to 27.73 %) (*43*). As carbohydrates increased, the energy density of beverages increased. Beverages based on powder had the highest energy value (403.84 kJ/100 g), followed by those based on Deglet Nour and syrup (379.87 and 377.48 kJ/100 g, respectively). The beverage made with Besser Helou had a lower energy density of 285.18 kJ/100 g. Although these drinks are not designed to replace milk, their energy density values and protein contents are within the proposed nutrient standards for plant-based beverages intended as milk alternatives, with a maximum of 355.64–418.4 kJ/100 g and a minimum protein mass fraction of 2.2 % (*44*). The increase of carbohydrate mass fractions (on dry mass basis) of different samples resulted in an increase in their TSS mass fractions (from (8.9±0.4) to (18.5±0.2) g/100 g), with the lowest value for the beverage based on Besser Helou and the highest for the beverage based on powder. Drinks made from Deglet Nour and syrup showed statistically similar values of (15.8±0.1) and (16.8±1.4) g/100 g, respectively. Additionally, it has been noted that the densities of the beverages increased, suggesting that the date ingredients may function as both thickeners and sweeteners. This effect was more pronounced in powder ((1054.8±0.8) g/L), with the highest dry matter content compared to fresh Deglet Nour ((1034.3±0.6) g/L), syrup ((1029.94±0.05) g/L) and Besser Helou ((1009.6±0.4) g/L). The titratable acidity of the drinks increased from (1.49±0.07) to (2.95±0.07) %, indicating that date ingredients enriched the beverages with organic acids. A decrease in pH between (5.07±0.06) and (5.97±0.12) was also observed, suggesting an acidifying effect of these products. However, the drinks remained in the low-acid food category (pH>4.5), requiring refrigerated storage if pasteurised (*43*). Water activity of the different beverages did not change with syrup or powder supplementation, but decreased with Deglet Nour and increased with Besser Helou supplementation.

#### Functional attributes of beverages

According to the data shown in Table 2, the phenolic composition of the almond beverage was similar to that of blanched almonds, with the notable appearance of the quercetin derivative hyperoside (quercetin-3-O-galactoside) and the absence of naringenin, luteolin and cirsiliol. Caffeic acid, which was not detected in the raw ingredients, was found in the beverages made from the combination of almonds with Deglet Nour, Besser Helou, and syrup, as well as in all residues.

The presence of this phenolic acid might be attributed to the decomposition of chlorogenic acids (CGA) into caffeic acid and quinic acid under the influence of pasteurisation. CGA are indeed temperature-sensitive. Increasing the temperature leads to intramolecular isomerisation and transesterification of these compounds, as well as their degradation. The diCGA degrade to the corresponding monoCGA and then to caffeic and quinic acid. The amount of each produced component varies with heating duration and temperature (*45*). Therefore, the presence of quinic acid in all produced drinks may be attributed not only to almonds but also to the breakdown of CGA originally present in each ingredient. The comparison of the phenolic composition of each beverage with the corresponding raw materials (almonds, date-based ingredient) revealed the appearance of new compounds: hyperoside in Besser Helou and Deglet Nour beverages, naringenin in Besser Helou beverage, naringin in Deglet Nour beverage, quercitrin (quercetin 3-O-glycoside derivative) in both Deglet Nour and syrup beverages, and *o*-coumaric acid and rutin (quercetin 3-O-glycoside derivative) in syrup-based beverage. These compounds are poorly soluble in water, so they could not be the result of the water extraction that occurred during the production of beverages. However, they possibly existed at low mass fractions in the raw materials and the large quantities of ingredients used for the preparation of drinks were sufficient to allow their detection.

The quantitative profiles of phenolics differed significantly between beverages. For example, the almond beverage contained the highest amounts of quinic acid and salvianolic acid. The Besser Helou-based beverage had the highest quantities of rosmarinic acid, hyperoside and naringin. The Deglet Nour-based beverage was the richest in caffeic acid, 1,3-di-O-caffeoylquinic acid, *o*-coumaric acid, luteolin and cirsiliol. The syrup-based beverage was the richest in chlorogenic acid, quercitrin and apigenin-7-O-glucoside. The highest mass fraction of quercetin was found in the powder-based beverage. Similar to the ingredients, the total phenolic acid content in beverages was higher than that of flavonoids. Combining date ingredients with almonds substantially increased the total flavonoid mass fraction in beverages, and consequently the total mass fraction of identified phenolics, except in the powder-based drink ((84.66±0.01) mg/100 g). This may be because it contained the least mass fraction of quinic acid and no caffeic acid compared to the other drinks. Deglet Nour was the ingredient that resulted in the highest mass fraction of total identified phenolics ((319.30±0.03) mg/100 g), followed by Besser Helou ((269.28±0.04) mg/100 g) and date syrup ((211.02±0.01) mg/100 g).

FRAP and DPPH assays revealed clear differences in the antioxidant capacity of the tested ingredients. The beverage enriched with date powder showed the strongest activity, with an exceptionally high FRAP value, expressed as Trolox equivalents (TE), ((39.56±0.06) mg/100 g) and similarly elevated DPPH scavenging capacity, expressed as TE, ((47.5±2.5) mg/100 g), significantly surpassing all other samples. The beverage based on date syrup also exhibited high antioxidant activity, clustering with the powder-based drink in the DPPH assay ((48.5±2.1) mg/100 g), but showing a more moderate reducing power in FRAP ((15.2±0.2) mg/100 g). Beverages enriched with Deglet Nour and Besser Helou showed intermediate antioxidant capacities. Almond beverage consistently had the lowest antioxidant activity in both assays.

When DPPH results were compared with values reported in the literature, it appeared that the incorporation of date fruits enabled the development of beverages with markedly higher antioxidant capacities than those fortified with other fruit- or plant-based ingredients. For instance, previous studies reported DPPH activities of (8.51±0.06), (12.22±0.08) and (9.0±0.06) mg/100 g for almond beverages supplemented with: (*i*) blended boiled carrot, honey and stevia, (*ii*) the same mixture with the addition of quinoa seeds, and (*iii*) banana juice, honey, stevia and oat powder, respectively. Notably, the almond mass fraction in these formulations (11–12 %) was comparable to that used in the present study (*7*).

Regardless of the quantitative aspect, almond and enriched beverages all contained potent phenolics recognised for their healing properties. Most of these compounds had antioxidant, antidiabetic, anticancer, antibacterial, antiviral, anti-aging, anti-nociceptive, anti-inflammatory and analgesic effects.

#### Colour characteristics of beverages

**Fig. S2:**
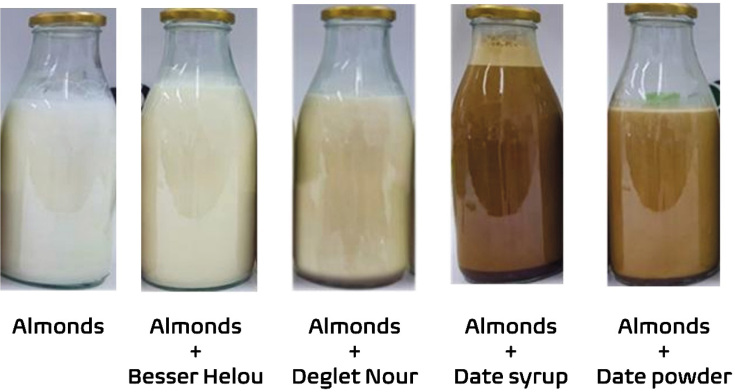
Visual aspect of beverages

The colour attributes of the beverages (Table 3) showed a clear colour difference between samples (Fig. S2). The almond drink was characterised by a white colour, while the beverage based on Besser was slightly yellowish. These beverages had the highest WI value and the lowest YI and BI values. The beverage based on Deglet Nour, which was brownish, showed statistically similar BI and YI values, but a lower WI. Those made with date powder (brown) and syrup (deep brown) had the highest YI and BI values, but differed in WI. These results indicated that certain pigments were transferred from dates and their products during processing and diffused into the aqueous phase, resulting in the formation of yellow and brown colours.

### Rheological properties of beverages

**Fig. 1:**
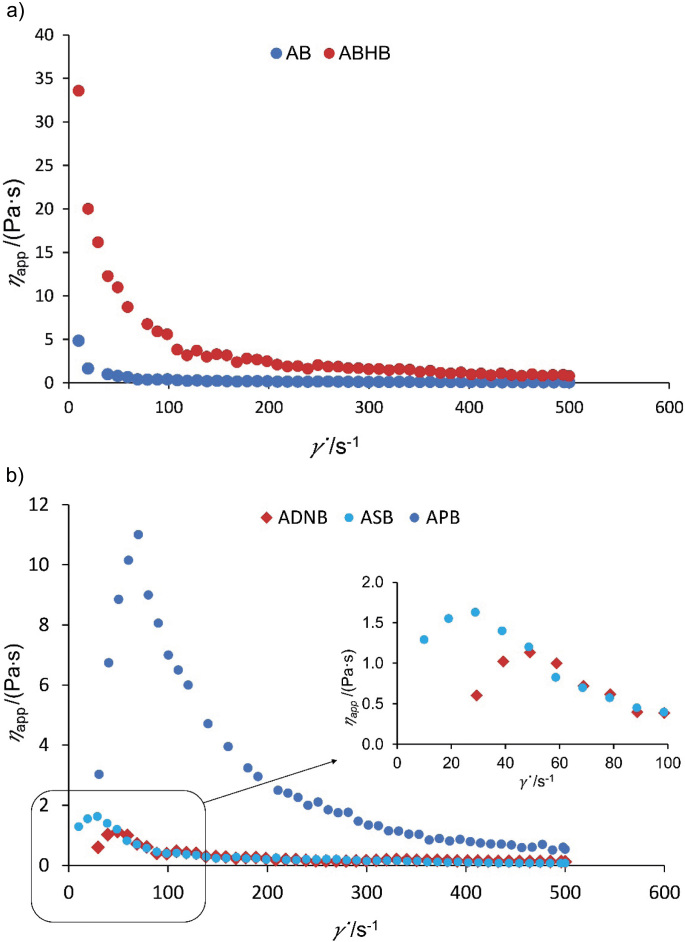
Flow curves of beverages based on: a) almonds (AB) and Besser Helou (ABHB), and b) Deglet Nour (ADNB), date syrup (ASB), and date powder (APB) showing apparent viscosity (*η*_app_/(Pa·s)) as a function of shear rate (*𝛾˙*/s^-1^). A magnified view of the low-shear rate region (upper right panel) highlights the shear thickening behaviour observed below approx. 80 s^−1^, followed by the onset of shear-thinning at higher shear rates

Rheological profile characterisation showed distinct flow behaviours among the five beverage formulations. Both the almond beverage and the Besser Helou-enriched beverage exhibited pronounced non-Newtonian shear-thinning behaviour, with apparent viscosity decreasing continuously as shear rate increased (Fig. 1a). This is typical of plant-based beverages, where suspended particles or polymeric structures are held together by weak forces that are progressively disrupted under stress (*46*).

In contrast, the beverages enriched with Deglet Nour, date syrup and date powder showed shear thickening behaviour at low shear rates, which transitioned into shear-thinning as the shear rate increased further (Fig. 1b). This may be explained by the high content of dietary fibre in Deglet Nour (*47*), which can form a gel-like network that resists deformation at low shear rates. This particular rheological pattern has been reported for polysaccharides such as lentinan and xanthan in water, curdlan in 0.01 M NaOH (*48*), as well as for apple and tomato pectins (*49*). It has been attributed to particle–particle collisions at low shear rates, which promote the formation of larger aggregates and result in increased viscosity as shear rate rises. At higher shear rates, however, the stronger shear forces disrupt these aggregates, causing the system to transition into shear-thinning, pseudoplastic behaviour (*48*). The rheological parameters derived from these flow curves are shown in Table 4. Although the almond beverage and the Besser Helou-enriched beverage exhibited similarly low flow behaviour indices (*n*≈0.025–0.027), indicating comparable shear-thinning responses, their consistency indices differed markedly, with the Besser Helou-enriched beverage (*K*=385.10 Pa·s^n^) being much more viscous than the almond beverage (*K*=29.06 Pa·s^n^). To characterise this specific rheological behaviour, a coefficient - referred to as the extent of shear thickening - has been proposed to quantify the degree of shear thickening observed at low shear rates. In addition, the *n* and *K* parameters of the power-law model are determined within the shear-thinning region. This coefficient corresponds to the ratio of the peak viscosity to the viscosity at the onset of shear thickening *(η*_top_*/η*_onset_) (*49*). These parameters, presented in Table 4, showed that the beverage based on date powder had the greatest extent of shear thickening (*η*_top_*/η*_onset_=3.63), likely due to high particle amount or stronger particle–particle interactions, as supported by its very high consistency index (*K*=430.74 Pa·s^n^), followed by the beverage enriched with Deglet Nour (*η*_top_*/η*_onset_=1.88) and syrup (*η*_top_*/η*_onset_=1.26). Overall, the contrasting behaviours highlight substantial differences in microstructure and particle interactions among the formulations. Shear-thinning beverages (based on almonds and Besser Helou) are expected to flow more easily under agitation, while the shear thickening beverages may present higher resistance at low shear but structural destabilization at higher deformation rates, with implications for mouthfeel, processing and stability.

**Table 4:** Power law constants describing the steadyshear rheological behaviour of beverages based on almonds (AB), Besser Helou (ABHB), Deglet Nour (ADNB), date syrup (ASB), and date powder (APB), evaluated only within the shear-thinning regions, along with the extent of shear thickening (*η*_top_/*η*_onset_)

Beverage	*K*/(Pa·s^n^)	*n*	R^2^	*η*_top_/*η*_onset_
AB	(29.1±2.0)^b^	(0.025±0.001)^b^	0.9986	-
ABHB	(385±2)^c^	(0.027±0.001)^b^	0.9764	-
ADNB	(22.1±1.0)^a^	(0.162±0.002)^d^	0.9676	(1.88±0.01)^b^
ASB	(29.0±1.0)^b^	(0.068±0.001)^c^	0.8879	(1.26±0.01)^a^
APB	(431±2)^d^	(0.001±0.001)^a^	0.9239	(3.63±0.01)^c^

### Physical stability of almond and enriched beverages

**Fig. S3:**
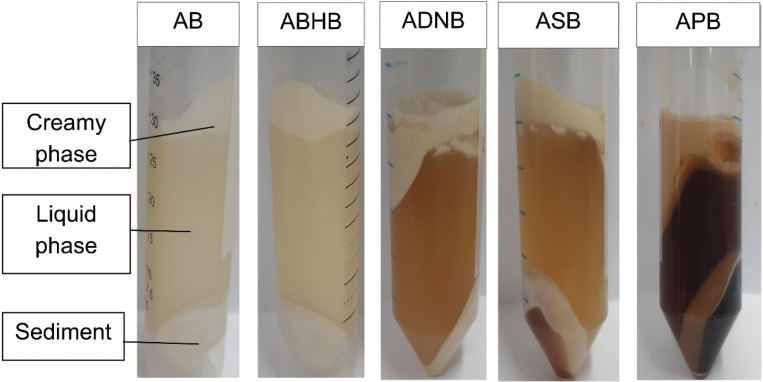
Different phases obtained after centrifugation of beverages based on almonds (AB), Besser Helou (ABHB), Deglet Nour (ADNB), date syrup (ASB), and date powder (APB)

**Fig. S4:**
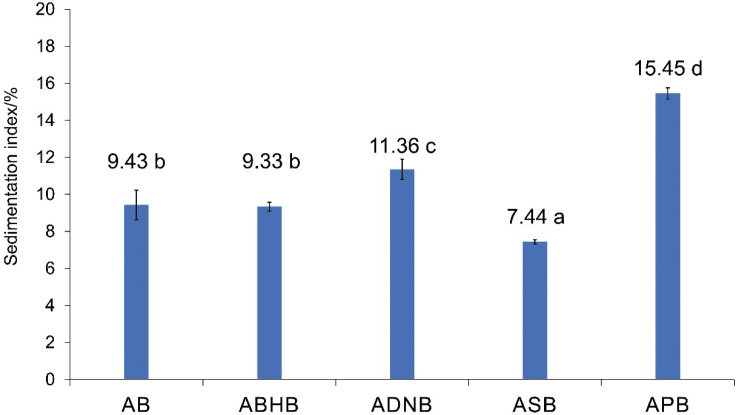
Sedimentation index of beverages based on almonds (AB), Besser Helou (ABHB), Deglet Nour (ADNB), date syrup (ASB), and date powder (APB). Different lower-case letters above the bars indicate statistically significant differences between samples at p<0.05

**Fig. S5:**
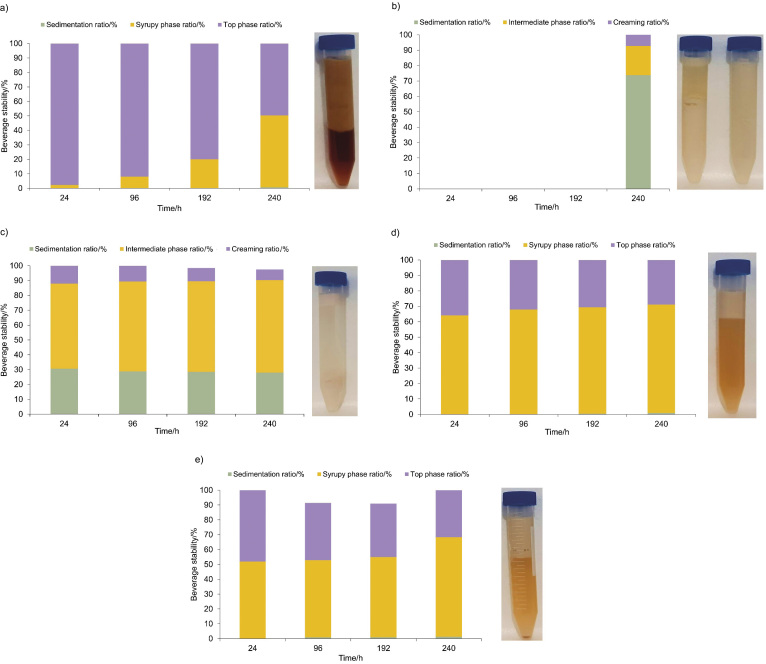
Stability of beverages based on: a) date powder, b) Besser Helou, c) almonds, d) Deglet Nour, and e) date syrup at 4 °C during 240 h

Although all formulated beverages exhibited a similar phase separation pattern after centrifugation (accelerated phase separation), characterised by a bottom sediment layer composed of the densest insoluble particles, an intermediate liquid phase, and an upper cream layer, as illustrated in Fig. S3, the extent of this separation differed between formulations, as reflected by the sedimentation index (Fig. S4). The beverage based on date powder had the highest sedimentation index (15.45), followed by the beverage enriched with Deglet Nour (11.36), while beverages based on almonds, Besser Helou and date syrup exhibited lower values (9.43, 9.33 and 7.44, respectively), indicating a lower tendency to sediment under centrifugal force. However, this trend did not strictly match the stability behaviour observed during storage at rest. Indeed, despite its high sedimentation index, the beverage based on date powder showed good visual stability over time, with delayed phase separation reaching a 50:50 ratio only after 240 h (Fig. S5a), while the beverage based on Besser Helou remained fully homogeneous throughout the entire storage period (Fig. S5b) and only showed phase separation at the end of the test, similarly to the control almond beverage (Fig. S5c), with the formation of a sediment layer at the bottom, an intermediate liquid phase, and an upper creaming layer. This apparent discrepancy can be explained by the fact that centrifugation mainly emphasises density differences and particle size effects, while stability at rest depends on prolonged interactions between sugars, fibre, proteins and lipids, as well as on the microstructural organisation of the beverage. In the beverage enriched with Besser Helou, the presence of Besser Helou, characterised by its white flesh, leads to the diffusion of particles with density, colour and optical properties close to those of almond particles, promoting better dispersion in the continuous phase and enhancing product homogeneity. For the beverage with added date powder, the soluble and insoluble fibre released from Deglet Nour powder during beverage processing likely contributed to water structuring and fat droplet entrapment, thereby improving resistance to phase migration and visual instability during storage. The beverages enriched with Deglet Nour (Fig. S5d) and date syrup (Fig. S5e) developed only two phases, namely a lower syrup-rich phase and an upper dense phase, while the appearance of a distinct sediment layer was minimal and only observed at the end of storage.

### Sensory profile and overall acceptability of beverages

**Fig. 2:**
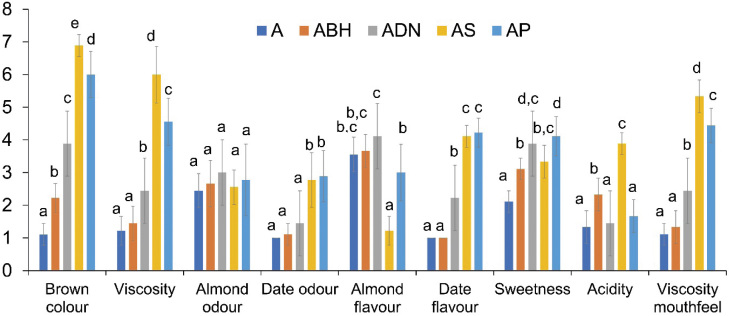
Descriptive sensory profiles of beverages based on almonds (A), Besser Helou (ABH), Deglet Nour (ADN), date syrup (AS) and date powder (AP)

The results in Fig. 2 show that the sensory profiles of the produced beverages differed significantly. The incorporation of date fruit ingredients, except for Besser Helou, increased the brown colour intensity and visual viscosity of the fortified beverages. The highest ratings for these attributes were given to the syrup-based beverage. All drinks showed the same low intensity of almond note. The note of dates was absent from the drinks based on almonds, Besser Helou and Deglet Nour fruits, and barely noticeable in the drinks based on powder and syrup. The flavour of almonds was predominant in the beverages based on almonds, Besser Helou and Deglet Nour fruits, while the flavour of dates was more pronounced in the drinks with powder and syrup. As expected, date ingredients increased the perception of sweet taste in all beverages, without exceeding the middle intensity range. Acidity was negligible in all products, except for the syrup-based beverage, which received the highest score. Mouthfeel viscosity followed the same trend as visual viscosity, with minimum levels in almond and Besser Helou beverages and maximum levels in syrup beverages.

**Fig. 3:**
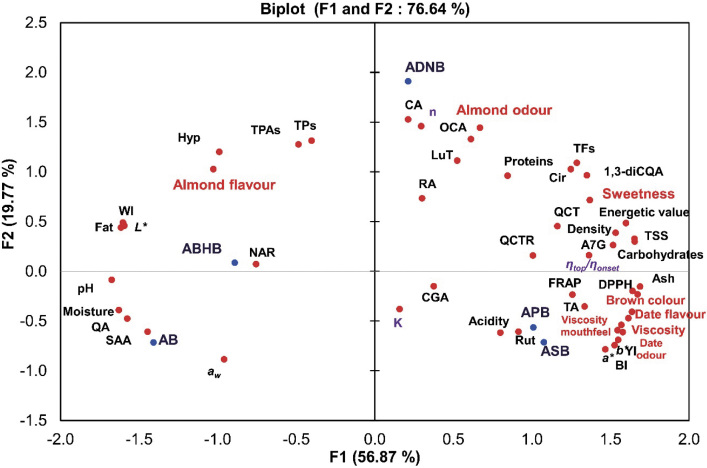
Principal component analysis of sensory attributes (red colour) and nutritional, physicochemical and antioxidant properties of beverages based on almonds (AB) and beverages fortified with Besser Helou (ABHB), Deglet Nour (ADNB), date syrup (ASB), and date powder (APB). QA=quinic acid, CGA=chlorogenic acid, CA=caffeic acid, 1,3-diCQA=1,3-di-O-caffeoylquinic acid, OCA=*o*-coumaric acid, RA=rosmarinic acid, SAA=salvianolic acid, Rut=rutin, Hyp=hyperoside, NAR=naringin, QCTR=quercitrin, A7G=apigenin-7-O-glucoside, QCT=quercetin, NRG=naringenin, LuT=luteolin, Cir=cirsiliol, TPAs=total phenolic acids, TFs=total flavonoids, TPs=total phenolics, TSS=total soluble solids, TA=titratable acidity, *a*_w_=water activity, L*=lightness, a*=redness, b*=yellowness, WI=whiteness index, YI=yellowness index, BI=browning index, *K*=consistency index, *n*=flow index, *η*_top_/*η*_onset_=extent of shear thickening, DPPH and FRAP=antioxidant activities

The PCA applied to sensory attributes and physicochemical properties (Fig. 3) explained 76.64 % of the variation in the data with the first two principal components (PC1=56.87 % and PC2=19.77 %). PC1 was associated positively with the attributes of brown colour, odour and flavour of dates, visual viscosity, mouthfeel viscosity and acidity, but negatively with almond flavour. Strong Pearson correlations were found between the attributes loaded positively on PC1, except for acidity, with DPPH antioxidant activity (0.920˂r˂0.975), ash content (0.935˂r˂0.988), and colour parameters: a* (0.924˂r˂0.978), b* (0.903˂r˂0.988), YI (0.948˂r˂0.998) and BI (0.947˂r˂0.991). These physicochemical properties were at higher values in the beverages obtained from the mixture of almonds with processed dates (powder and syrup), which were also loaded positively on PC1. A strong positive correlation was also observed between FRAP and DPPH assays (r=0.774), indicating good consistency between the two antioxidant evaluation methods. The attribute of almond flavour was relatively strongly correlated with fat content (r=0.817) and colour parameters: L* (r=0.836) and WI (0.823), which were associated with the beverages obtained from almonds and the combination of almond and Besser Helou. PC2 was associated positively with almond odour and sweetness. The sweet taste was highly correlated with density (r=0.975), energy value (r=0.950), TSS (0.917), carbohydrate content (r=0.908) and the extent of shear thickening (r=0.849), and was associated with the beverage based on date powder. Acidity was more closely correlated with the contents of rutin (r=0.930) and chlorogenic acid (r=0.819), and was linked to the beverage based on date syrup. An intriguing correlation between almond odour and luteolin was noted (r=0.920). This flavonoid is renowned for its ability to improve olfactory memory and has been found to enhance olfactory dysfunction and memory in individuals with long COVID and chronic olfactory loss when combined with palmitoylethanolamide (*50*).

**Fig. 4:**
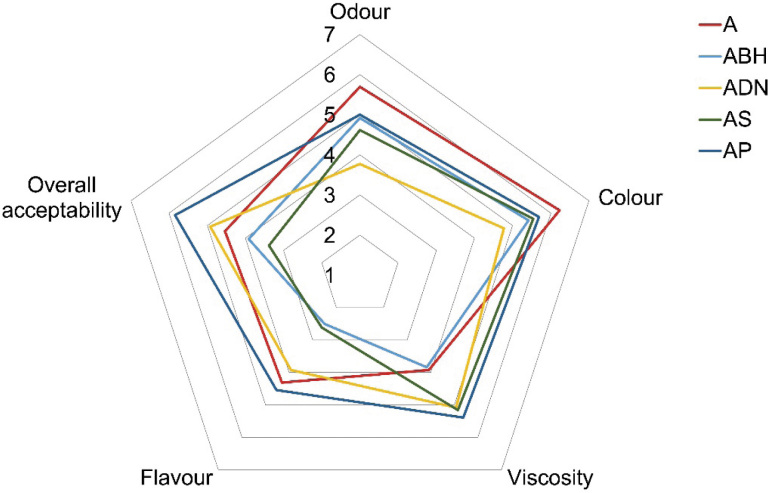
Hedonic evaluation of beverages based on almonds (A), Besser Helou (ABH), Deglet Nour (ADN), date syrup (AS) and date powder (AP)

Fig. 4 shows the results obtained from hedonic ratings. The almond-based beverage showed the highest mean liking scores for odour and colour attributes, 5.69 and 6.23, respectively, while the Deglet Nour beverage showed the lowest scores (3.77 and 4.77, respectively). The five beverages showed similar results for the viscosity attribute. For the flavour attribute, the highest liking score was given to the powder-based drink (4.54), followed by the almond and Deglet Nour drinks, 4.31 and 3.92, respectively. The beverages based on Besser Helou and syrup received the lowest scores, 2.50 and 2.62, respectively. Overall acceptance was highest for powder-based beverages (5.85) and lowest for syrup-based beverages (3.39).

### Nutritional and functional characterisation of beverage residues

#### Compositional and physicochemical attributes of residues

Table 1 shows the compositions of the produced residues. The results indicate that, after direct extraction of beverages, they contained more than 50 % moisture and had high, comparable water activity values ranging from 0.9504 to 0.9701. Similar moisture mass fractions have been reported for by-products from the preparation of almond ((62.2±0.2) %), coconut ((55.0±0.2) %) and oat ((60.6±0.1) %) drinks (*51*). Notably, the most water-rich residue ((71.9±0.2) %) was derived from Besser Helou, the wettest ingredient. As these co-products are generally preserved and valued in dried form, they were dried until reaching a water activity of 0.6. The almond residue had the lowest carbohydrate mass fraction ((10.65±0.04) %), but the highest mass fractions of ash ((2.74±0.03) %), fat ((58.39±0.01) %) and protein ((22.47±0.03) %), making it the most calorie-dense by-product. Combining date ingredients with almonds decreased the mass fraction of fat in residue ((24.86-41.93) %) and protein ((8.4-14.4) %), while significantly increasing carbohydrate mass fraction ((36.52-53.0) %). This increase in carbohydrates could mostly be due to insoluble fibre retention. Indeed, the TSS mass fractions of the used by-products ((7.2-13.2) g/100 g) were low, although higher than that of the almond-based one ((2.4±1.6) g/100 g). All investigated by-products had comparable ash mass fractions ((2.3±0.1)-(2.74±0.03) %), except for date powder, which had the lowest value ((1.8±0.4) %).

Since combined date and almond residues had lower fat and protein contents than almond residues, they showed lower but considerable caloric values, ranging from 1991 (Deglet Nour) to 2347 kJ/100 g (syrup). The residues based on almonds, Deglet Nour and syrup had higher pH values than those based on Besser Helou and date powder. All samples showed comparable TA values, except for date powder residue, which had the highest value, indicating that it retained more organic acids than the others. Some studies have investigated the incorporation of such co-products in bakery products as replacements for wheat or almond flour. It has been reported that partial substitution of wheat flour with dried almond residue improves the nutritional value of biscuits due to its richness in fibre, protein and fat, with limited impact on the sensory properties of the final products (*52*). Innovative residue processing methods, such as fermentation and ultrasonication, applied before replacing wheat flour with almond by-product in white bread, showed that including 20 % fermented almond residue resulted in the highest overall acceptance of final products, while the ultrasonicated one significantly decreased the acrylamide amount in bread (*51*).

#### Functional attributes of residues

All residues retained considerable quantities of bioactive compounds and showed notable antioxidant activity (Table 2). As observed in beverages, some phenolics not present in the raw materials appeared in the by-products. These included *trans*-ferulic acid, detected only in the date powder residue, and hyperoside. Caffeic acid, absent from almond and powder beverages, was found in the corresponding by-products as well as in Besser Helou, Deglet Nour and syrup residues. This supports the previously proposed hypothesis that caffeic acid forms as a result of chlorogenic acid breakdown during heat treatment. Each by-product was characterised by the predominance of one or more phenolic compounds. For example, the almond-based residue contained the highest mass fractions of quinic, chlorogenic and salvianolic acid. The Besser Helou-based residue was richest in 3-di-O caffeoylquinic acid, *trans*-ferulic acid, *o*-coumaric acid, hyperoside, naringin, naringenin and cirsiliol. The Deglet Nour-based by-product had the highest mass fraction of luteolin. The residue based on syrup was characterised by the abundance of rosmarinic acid and apigenin-7-O-glucoside, while that based on powder was the richest in caffeic acid, quercetin and quercitrin.

Generally, the almond-based residue was distinguished by the predominance of quinic acid among the phenolic acids and quercetin among the flavonoids, while the date-based residues were characterised by the prevalence of caffeic acid ((53.439±0.001)–(67.899±0.001) mg/100 g) and luteolin ((5.517±0.001)–(45.836±0.002) mg/100 g). The by-product derived from almonds contained lower mass fraction of flavonoids ((6.81±0.03) mg/100 g) and more phenolic acids ((294.7±0.6) mg/100 g) than those derived from date fruits, and consequently more phenolics than the other samples ((301.5±0.6) mg/100 g). Among the by-products based on date ingredients, those derived from Deglet Nour had the highest mass fractions of total identified phenolics ((228.194±0.003) mg/100 g) and total flavonoids ((59.810±0.003) mg/100 g). Although the almond-based residue had the highest phenolic mass fraction, it had the lowest antioxidant capacity. The DPPH value, expressed as TE, was (27.6±3.0) mg/100 g, consistent with the values (on dry mass basis) reported by Duarte *et al.* (*53*) of 0.20–0.23 mg/g for almond by-products dried at 60 and 70 °C, respectively. The by-products based on Deglet Nour, syrup and date powder had comparable DPPH values of (52.9±0.6), (50.6±0.5) and (53.3±0.3) mg/100 g, respectively, while the residue based on Besser Helou showed a lower value of (46.0±2.5) mg/100 g. A significant variation in FRAP values was observed, with the date powder residue showing the highest value ((52.6±5.0) mg/100 g) and Besser Helou the lowest ((12.3±0.1) mg/100 g), suggesting that the bioactive compounds responsible for high DPPH and FRAP values were largely preserved in the date powder.

#### Colour characteristics of residues

**Fig. S6:**
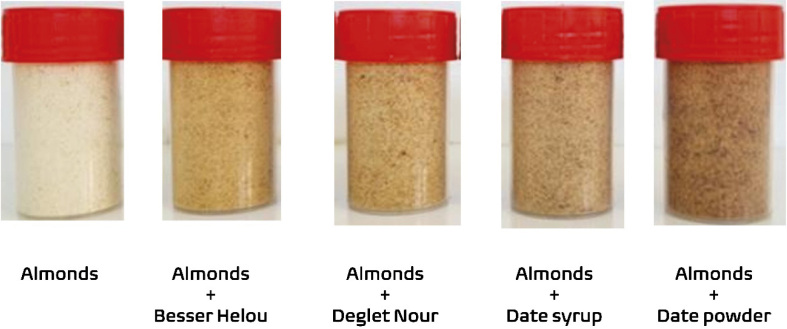
Visual aspect of residues

Fig. S6 shows the colours of the produced residues. The almond-based residue was white, with the highest WI and the lowest YI and BI values (Table 3). The residues obtained from dates and their derived products showed different shades of brown. The Besser Helou-based residue was light yellowish brown, with the highest WI and the lowest BI and YI among the date-based residues. The residue obtained from date powder was a deeper brown than that from Deglet Nour and the syrup, thus displaying the lowest WI and the highest BI.

## CONCLUSIONS

This study highlights the strong potential of date fruits as natural ingredients for the fortification of almond-based beverages. The incorporation of date-derived products significantly enhanced the antioxidant properties of the formulations, as shown by increased FRAP and DPPH values. The positive correlation observed between these two assays further confirmed the consistency and reliability of the antioxidant improvement attributed to date constituents, which are rich in phenolics, flavonoids, and other bioactive compounds. Rheological characterisation revealed that all fortified beverages exhibited non-Newtonian behaviour, with certain date-enriched formulations displaying distinctive shear thickening behaviour at low shear rates followed by shear-thinning at higher shear rates. This flow profile, likely related to the presence of date polysaccharides and pectic substances, may contribute positively to mouthfeel and texture, offering sensory and processing advantages in beverage applications. In terms of physical stability, date fortification influenced sedimentation and phase separation. While some formulations exhibited clear separation into syrup and serum phases during storage, others maintained a more homogeneous structure for extended periods, demonstrating improved colloidal stability. This suggests that date ingredients not only provide nutritional benefits but also act as natural stabilising agents by modifying the continuous phase and particle interactions within the beverage matrix. Importantly, this work provides the first comprehensive evaluation of the combined effects of date fruit ingredients on the nutritional, rheological and stability attributes of fortified almond beverages. The findings open new perspectives for the development of clean-label, plant-based functional drinks with enhanced health benefits and tailored physicochemical properties, using locally available agro-resources such as date fruits.

## SUPPLEMENTARY MATERIALS

Supplementary materials are available at: https://www.ftb.com.hr/images/pdfarticles/2026/April-June/FTB-64-205-S1.pdf.
